# P-188. The Potential Impact of Vaccination on Clade I Mpox Cases in the Democratic Republic of Congo, and Risk of Importation to the United States from International Travel

**DOI:** 10.1093/ofid/ofae631.392

**Published:** 2025-01-29

**Authors:** Zainab Riyaz, Peter Mortensen, Samuel Cutler, Thomas Maguire, Matt Linley, Jacqueline Buchanan

**Affiliations:** Airfinity, London, England, United Kingdom; Airfinity, London, England, United Kingdom; Airfinity, London, England, United Kingdom; Airfinity, London, England, United Kingdom; Airfinity, London, England, United Kingdom; Airfinity, London, England, United Kingdom

## Abstract

**Background:**

The mpox public health emergency of international concern that emerged in 2022, driven by clade IIb mpox infections in high-income nations, was declared over by the WHO in May 2023, while the more virulent clade I mpox, continues to spread endemically in Central Africa. In November 2023, the Democratic Republic of Congo (DRC) reported its highest ever annual clade I mpox cases, reaching 12,569 with a 4.6% case fatality rate (CFR). The DRC lacks rollout of approved mpox vaccines but has two vaccine trials among healthcare personnel ongoing. The US saw a 78% increase in clade IIb incidence in the first 11 weeks of 2024 compared to the same period in 2023. Only 25% of the eligible US population has completed the two-dose vaccine regimen; waning immunity may leave the population more susceptible to clade I infection should it be imported. Travel is increasing from the DRC and the Congo to the US, particularly within the past year, highlighting the need for enhanced surveillance and vaccination.

**Figure:**
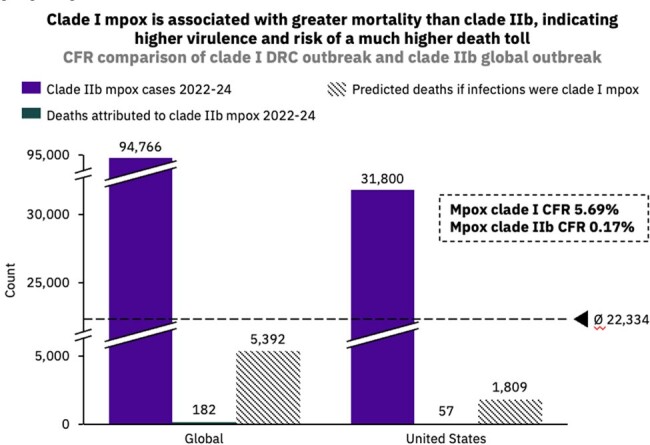

**Methods:**

Three vaccination scenarios for the DRC, beyond the baseline of no vaccines received, were built: 1) vaccinating the public who have not received the smallpox vaccine, 2) vaccinating the high-risk GBMSM (gay, bisexual and other men who have sex with men) population aged 18-40 years and 3) vaccinating children, another high-risk group. Direct and indirect international travel volumes from the DRC to the US were analysed and forecasted to assess risk of the introduction of clade I in the US.

**Figure:**
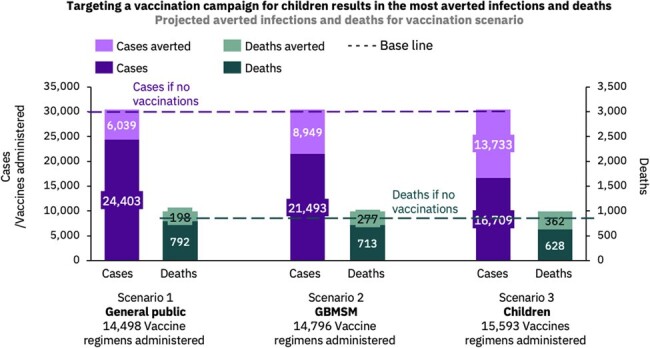

**Results:**

Our data suggest that if clade I mpox spreads similarly to clade IIb in 2022, the expected death toll could be > 5000 deaths globally (CFR 5.69%) and ∼ 1800 in the US. A GBMSM-targeting vaccine campaign could avert ∼ 9000 infections and > 250 deaths in the DRC. The number of travellers from the DRC to the US is forecasted to reach a high of > 5600 passengers between April-August 2024, with indirect travel also posing a risk. The top three countries with travel to the US include the UK, France and India; France sees the highest levels of travel from the Congo and the DRC, increasing the risk of onward clade I transmission if an introduction were to occur.

**Figure:**
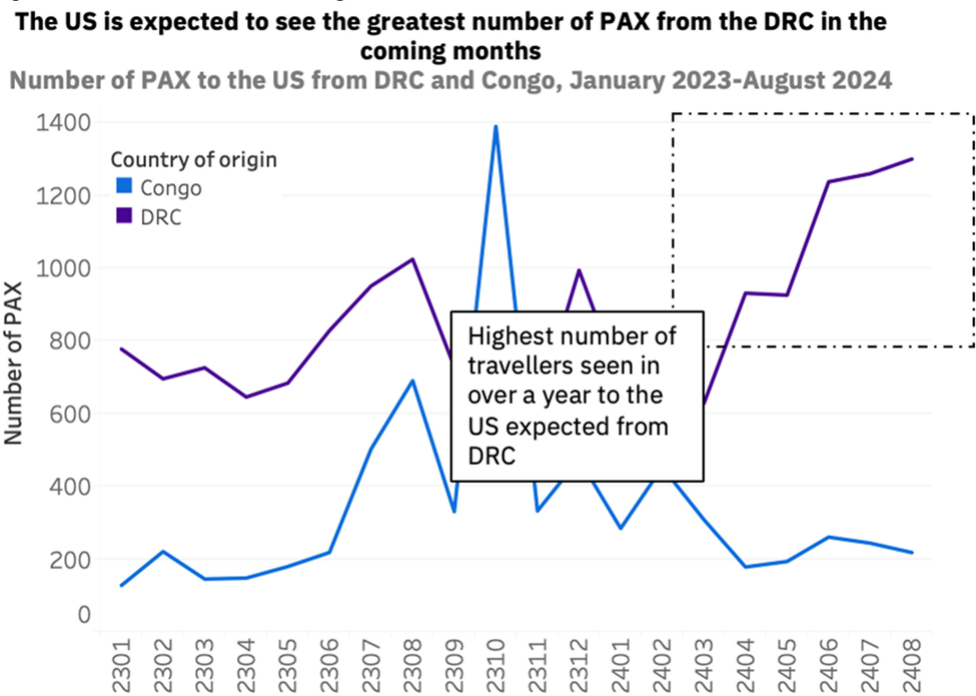

**Conclusion:**

Vaccinating the GBMSM in the DRC could significantly reduce infection rates and reduce the clade I risk posed to nations, such as the US, via international travel.

**Figure:**
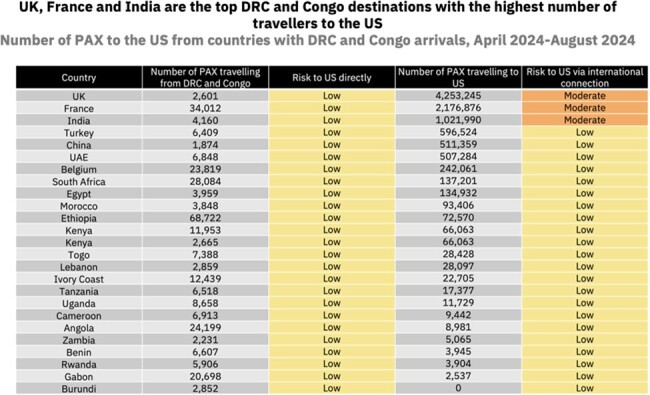

**Disclosures:**

**All Authors**: No reported disclosures

